# Electrical and thermal characterisation of liquid metal thin-film Ga$_2$O$_3$–SiO$_2$ heterostructures

**DOI:** 10.1038/s41598-023-30638-4

**Published:** 2023-03-01

**Authors:** Alexander Petkov, Abhishek Mishra, Mattia Cattelan, Daniel Field, James Pomeroy, Martin Kuball

**Affiliations:** 1 HH Wills Physics Laboratory, University of Bristol, Bristol, BS8 1TL UK; 2 School of Chemistry, University of Bristol, Cantocks Close, Bristol, BS8 1TS UK; 3 Department of Chemical Sciences, University of Padova, Via Marzolo 1, 35131 Padova, Italy

**Keywords:** Electronic devices, Surfaces, interfaces and thin films, Electronic properties and materials

## Abstract

Heterostructures of Ga$_2$O$_3$ with other materials such as Si, SiC or diamond, are a possible way of addressing the low thermal conductivity and lack of p-type doping of Ga$_2$O$_3$ for device applications, as well as of improving device reliability. In this work we study the electrical and thermal properties of Ga$_2$O$_3$–SiO$_2$ heterostructures. Here, thin-film gallium oxide with thickness ranging between 8 and 30 nm was deposited onto a silicon substrate with a thermal oxide by means of oxidised liquid gallium layer delamination. The resulting heterostructure is then characterised by means of X-ray photoelectron spectroscopy and transient thermoreflectance. The thin-film gallium oxide valence band offset with respect to the SiO$_2$ is measured as 0.1 eV and predicted as $-2.3$ eV with respect to diamond. The thin-film’s out-of-plane thermal conductivity is determined to be 3 ±0.5 Wm$^{-1}$ K$^{-1}$, which is higher than what has been previously measured for other polycrystalline Ga$_2$O$_3$ films of comparable thickness.

## Introduction

Gallium oxide is an ultra-wide band gap material (4.8 eV for its $\beta$ polymorph^1^) that has attracted a lot of attention for power electronics in recent years. Its breakdown electric field is predicted to be around 8 MV cm$^{-1}$,^2^ significantly higher than the 2.6 MV cm$^{-1}$ and 3.3 MV cm$^{-1}$ for SiC and GaN respectively, which are established materials for power electronics applications^3^. Gallium oxide offers the potential for ultra-high voltage power device technology, even exceeding 10 kV. This, along with its high Baliga figure of merit and low cost substrates due to the availability of melt-grown Ga$_2$O$_3$, has made gallium oxide an attractive material for power electronic devices for use in various high-voltage applications, including power conversion, electric vehicles, data centres^4,5^. There has also been significant interest in the fabrication of 2D thin-film gallium oxide for potential 2D material-based applications, such as gas sensing^6^, water-splitting solar cells^7^ and even wearable electronics^8^.

Thermal transport is one of the main challenges for Ga$_2$O$_3$-based devices. The most thermodynamically stable phase of gallium oxide, $\beta$-Ga$_2$O$_3$, has a relatively low thermal conductivity, which is also anisotropic, ranging between 11 Wm$^{-1}$ K$^{-1}$ and 27 Wm$^{-1}$ K$^{-1}$ depending on crystallographic direction^9,10^. To put this in perspective, the relevant values for SiC and GaN are about an order of magnitude higher at 420 Wm$^{-1}$ K$^{-1}$ and 160 Wm$^{-1}$ K$^{-1}$ respectively^11^. For any potential device, the low thermal conductivity of the semiconductor may lead to device failure under operation due to poor thermal dissipation. A possible solution to this is the integration of Ga$_2$O$_3$ with a high thermal conductivity material/substrate. Numerous approaches have been reported including integration with SiC via wafer bonding, where temperature rise has been predicted to reduce by up to 30% for a bottom side cooling scheme^12^. Another issue with Ga$_2$O$_3$ is its poor hole mobility, which together with the lack of suitable shallow acceptors, makes Ga$_2$O$_3$-based bipolar or p-type devices so far impossible^13^. A p-n junction, however, can be established by integration of n-type Ga$_2$O$_3$ with a p-type material, which has been accomplished with p-doped nickel oxide for the purpose of diodes with tuneable electrical and optical properties^14^, as well as p-doped GaN for self-powered photodetectors^15^. Furthermore, modelling showed that a p-n Ga$_2$O$_3$-diamond superjunction would lead to approximately 60% reduction in temperature rise under operation^16^. It is necessary to know the band alignment across the heterojunction to design efficient devices of this type. Note that local stoichiometric inhomogeneities in Ga$_2$O$_3$ have been shown to affect core and valence states in the material^17^. In fact, the valence band offset of Ga$_2$O$_3$ with silicon has been reported to vary for different gallium oxide polymorphs, ranging from -2.9 eV (for $\epsilon$-Ga$_2$O$_3$) to -3.7 eV (for $\kappa$-Ga$_2$O$_3$)^18^. Because of this, we may expect different electronic properties from different amorphous/polycrystalline Ga$_2$O$_3$ samples, such as the one examined here.

In this work we investigate the electrical and thermal properties of a Ga$_2$O$_3$-based heterointerface, realised through deposition of thin films of Ga$_2$O$_3$ onto silicon with thermal oxide. The deposition method used is based on the exfoliation of thin-film gallium oxide from liquid gallium - a recently proposed technique to realise 2D metal oxides^19^. Silicon has a thermal conductivity of about 130 Wm$^{-1}$ K$^{-1}$^20^, significantly higher than Ga$_2$O$_3$ and so is a potential material for thermal management of Ga$_2$O$_3$-based devices. We obtain values for the valence band offset of the deposited gallium oxide with SiO$_2$, its out-of-plane thermal conductivity and thermal boundary resistance to the silicon substrate. This data can also be used predictively to assess the thin-film gallium oxide viability for use in tandem with other high thermal conductivity substrates, such as diamond.

## Methods

**Figure 1:**
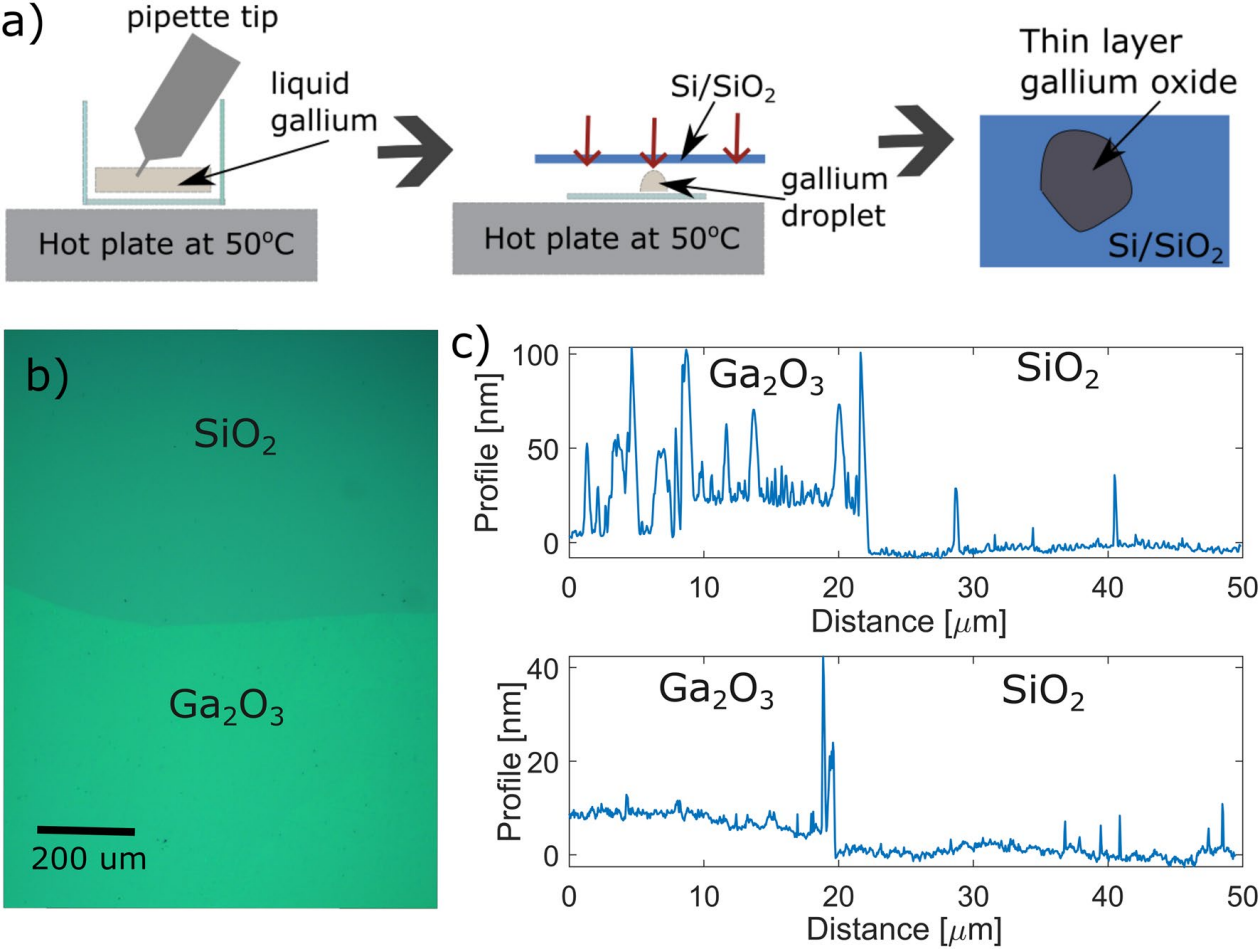
(**a**) Schematic of the exfoliation method - a liquid gallium droplet is isolated and its passivation oxide is directly transferred onto a substrate. (**b**) Microscope image of the Ga$_2$O$_3$ film deposited on thermally oxidised Si substrate after annealing at 250$^{\circ }$C for 1 hour. (**c**) Atomic Force Microscopy linescans taken across thin-film oxide to substrate edges in two different areas.

Pure gallium has a melting point slightly above room temperature - at 29$^{\circ }$C. When exposed to air, the surface of the liquid metal is spontaneously oxidised due to a low Gibbs Free Energy for the formation of Ga$_2$O$_3$^21^. This passivating oxide layer is up to a few nanometres thick and with a large chemical potential gradient at the interface between the liquid core and oxide layer^22^. Because of this van der Waals forces have been found sufficient to detach this oxide layer from the bulk and adhere it to a separate substrate^23^. A gallium pellet is taken and heated on a hot plate to 50$^{\circ }$C, i.e. above its melting temperature. A pipette tip is then used to pick up a liquid gallium droplet, which is in turn placed on a glass slide, kept in liquid form on the hot plate. The thin film of Ga$_2$O$_3$ or oxide skin is then put in contact with a B-doped Si substrate with thermal oxide, resulting in large area transfer of Ga$_2$O$_3$ film, as illustrated in Fig. 1a. Excess gallium is cleaned off by rinsing the sample in heated ethanol. The sample is then annealed in oxygen at 250 $^{\circ }$ C for 1 h. This step has been suggested to aid in stabilising the stoichimometry of the deposited Ga$_2$O$_3$ film^19^. A microscope image of the layer post annealing is shown in Fig. 1b; the boundary between the substrate and thin-film oxide is evident. An Agilent 5420 Atomic force microscope (AFM) was used in tapping mode, confirming that the layers prepared had a thickness ranging from 8 to 30 nm, shown in Fig. 1c. The extracted profile for a thicker sample is visibly uneven. This is likely due to an overlapping of several oxidation layers.

In order to measure the out-of-plane thermal conductivity of the Ga$_2$O$_3$, transient thermoreflectance (TTR) was used. This technique uses a nanosecond laser heating pulse and a continuous wave probe laser to measure the transient thermal response^24^. A frequency tripled 10 ns 355 nm Nd:YAG pump laser with a 30 kHz repetition rate and a spot diameter of 85 $\mu$m was used to heat up the sample surface and a 532 nm probe laser with spot size of about 2 $\mu$m was used to measure the induced transient reflectivity change. 10 nm of chromium and 100 nm of gold were thermally evaporated on the sample surface prior to the measurement, acting as a transducer. More details about the experimental TTR setup used here are given in Yuan et al.^25^. It should be noted that the probing depth of this TTR setup does not exceed 10 microns, and has lower sensitivity for layers under 100 nm^26^. Because of this, for the purpose of said measurements we choose to investigate the deposition area with the largest thickness (30 nm). Thermoreflectance transients were recorded for the Ga$_2$O$_3$, as well as on the bare SiO$_2$ as a reference, and an analytical model outlined in Yuan et al. was used to fit the thermal conductivities across different layers onto the data^25^.

High resolution X-ray photoelectron spectroscopy (XPS) was used to measure the valence band alignment of the Ga$_2$O$_3$ film to the substrate using a monochromatic Al k$\alpha$ (h$\nu$ = 1486.7 eV) excitation source with a pass energy of 50 eV. Information about the energetics of core levels and valence band maxima were extracted. The valence band offset of Ga$_2$O$_3$ with respect to SiO$_2$ is given as^28^

1 $$\begin{aligned} \Delta E_V= \left( E^{Ga_2O_3}_{Ga 3d}-E^{Ga_2O_3}_V\right) -\left( E^{SiO_2}_{Si 2p}-E^{SiO_2}_V\right) -\left( E^{Ga_2O_3-SiO_2}_{Ga 3d}-E^{Ga_2O_3-SiO_2}_{Si 2p}\right) , \end{aligned}$$

where $E_V^{Ga_2O_3}$ and $E^{SiO_2}_V$ denote the valence band energies for the two materials - Ga$_2$O$_3$ and SiO$_2$, respectively, $E^{SiO_2}_{Si 2p}$ and $E^{Ga_2O_3}_{Ga 3d}$ denote the energies of the core levels Si 2p and Ga 3d in the spectra taken solely from SiO$_2$ and Ga$_2$O$_3$, respectively, while $E^{Ga_2O_3-SiO_2}_{Ga 3d}$ and $E^{Ga_2O_3-SiO_2}_{Si 2p}$ denote the the energies of the two core levels as measured across the Ga$_2$O$_3$-SiO$_2$ interface. XPS has a low probing depth that rarely exceeds few tens of nanometres, however, due to the thin-film nature of the gallium oxide deposition, any measurement taken from the Ga$_2$O$_3$ film is expected to probe through the interface and into the SiO$_2$ layer. Because of this, values for $E_V^{Ga_2O_3}$ and $E^{Ga_2O_3}_{Ga 3d}$ cannot be reliably determined from our data, and a standard value for the term $E^{Ga_2O_3}_{Ga 3d}$-$E_V^{Ga_2O_3}$=17 eV is used instead^27^. For measurements, an area on the sample with gallium oxide deposition of 8 nm thickness was chosen, from which $E^{Ga_2O_3-SiO_2}_{Ga 3d}$ and $E^{Ga_2O_3-SiO_2}_{Si 2p}$ data was extracted. An area on the sample without deposition was also chosen for the estimation of $E^{SiO_2}_V$ and $E^{Ga_2O_3-SiO_2}_{Si 2p}$^29^. The peak positions for Ga 3d and Si 2p were estimated from the data via Gaussian fitting.

## Results and discussion

**Figure 2:**
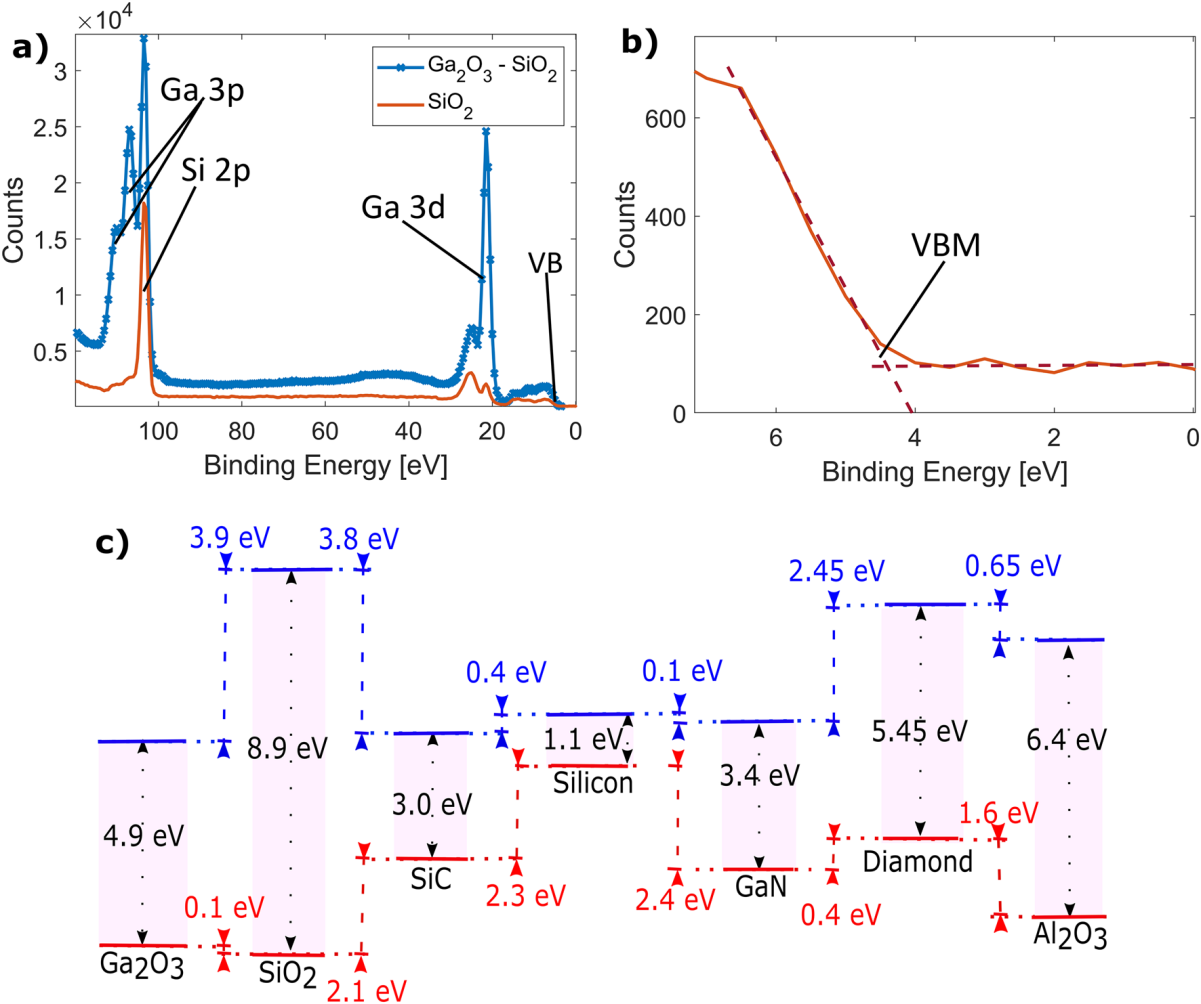
XPS energy spectra recorded from the (**a**) SiO$_2$ and Ga$_2$O$_3$ film on SiO$_2$; a zoom into the valence band region for the SiO$_2$ is shown separately in (**b**), where the intersect of dashed lines is used to identify the valence band maximum (VBM); (**c**) shows a diagram of the band alignment of the Ga$_2$O$_3$ film to the SiO$_2$ and extended to other materials. A band gap of 4.9 eV is assumed for our Ga$_2$O$_3$ film to determine conduction band offsets.

The recorded XPS spectra from the SiO$_2$ and deposited Ga$_2$O$_3$ on SiO$_2$ can be seen in Fig. 2a in red and blue respectively. Both data sets were rigid-shifted by 2.3 eV so that the Si 2p peak from the SiO$_2$ ($E^{SiO_2}_{Si 2p}$) spectrum is apparent at 103.3 eV as is standard^30^. The Si 2p peak is also visible in the Ga$_2$O$_3$-SiO$_2$ spectrum, although is slightly obscured by several overlapping Ga 3p peaks. On the Ga$_2$O$_3$-SiO$_2$ spectrum the Ga 3d peak is apparent at 21.5 eV with the additional peak at about 25 eV being related to oxidation - a characteristic feature of a Ga$_2$O$_3$ spectrum^31^. These peaks are also visible in the spectrum obtained from the SiO$_2$, though with significantly lower intensity, likely appearing due to residual traces of gallium from the deposition. The SiO$_2$ XPS spectrum in the close vicinity of the valence band maximum is seen in Fig. 2b. The value for the valence band energy is taken as the intercept of two linear fits around the points of steepest increase, determined as 4.4 eV.

**Table 1:** Table of biding energies used for valence band offset determination.

	Ga$_2$O$_3$	SiO$_2$	Ga$_2$O$_3$/SiO$_2$
Ga 3d	20.25 eV*		21.5 eV
Si 2p		103.3 eV	103.5 eV
VBM	3.23 eV*	4.4 eV	

$^*$Values taken from Huan et al.^27^.

The obtained 0.12 eV valence band offset of Ga$_2$O$_3$ with respect to SiO$_2$ is shown schematically in Fig. 2c. The binding energy values used for calculating said offset using (1) from the experimental data are given in Table 1. For the purpose of visualisation and conduction band offset discussion, we are taking a band gap value for the thin film deposited here equal to 4.9 eV (equal to the band gap of standard $\beta$-Ga$_2$O$_3$). This is consistent with high resolution transmission electron microscopy (HRTEM) characterisation on films deposited under identical conditions, identified as polycrystalline $\beta$-Ga$_2$O$_3$^23^. We also note that $\beta$-Ga$_2$O$_3$ is the thermodynamically most stable gallium oxide polymorph, with the second most stable - $\kappa$-Ga$_2$O$_3$ also having a band gap of 4.9 eV^18^. Taking the silicon oxide band gap as 8.9 eV^32^, that results in a conduction band offset of -4.0 eV for our thin film Ga$_2$O$_3$ with respect to SiO$_2$. Comparing to reported values in the literature, considering a valence band offset of 4.4 eV between Si to SiO$_2$^33^, the here obtained valence band offset of Ga$_2$O$_3$ to Si $\Delta E_V$ would be -4.3 eV, with a conduction band offset of 0.5 eV. For comparison, a value of -3.5 eV was reported for the valence band offset in a PLD $\beta$-Ga$_2$O$_3$-Si interface (with a conduction band offset of -0.2 eV), showing a significant difference between the pure $\beta$ phase and the film deposited here^34^. The change of sign between the two conduction band offsets implies that while a $\beta$-Ga$_2$O$_3$-Si junction has type I alignment, the Ga$_2$O$_3$ film deposited in this work would have a type II alignment to silicon. Figure 2c also shows predicted band alignment of the deposited thin-film Ga$_2$O$_3$ to GaN, SiC, Al$_2$O$_3$ and diamond, based on the measured band alignment of GaN with respect to SiO$_2$ and SiC^35^, GaN with respect to Al$_2$O$_3$^36^, and GaN with respect to diamond^37^. We thus estimate the valence band offset of the thin-film Ga$_2$O$_3$ to diamond as $-2.3$ eV, with a predicted conduction band offset of $-2.85$ eV. This alignment provides significant energetic barriers for minority carriers across a potential n-type Ga$_2$O$_3$ to p-type diamond heterojunction—about 0.8 eV higher than in PLD $\beta$-Ga$_2$O$_3$. This also correlates to a higher breakdown field in a potential Schottky barrier diode, such as the one proposed by Mishra et al., using a Ga$_2$O$_3$–Al$_2$O$_3$-diamond superjunction^16^.

**Figure 3:**
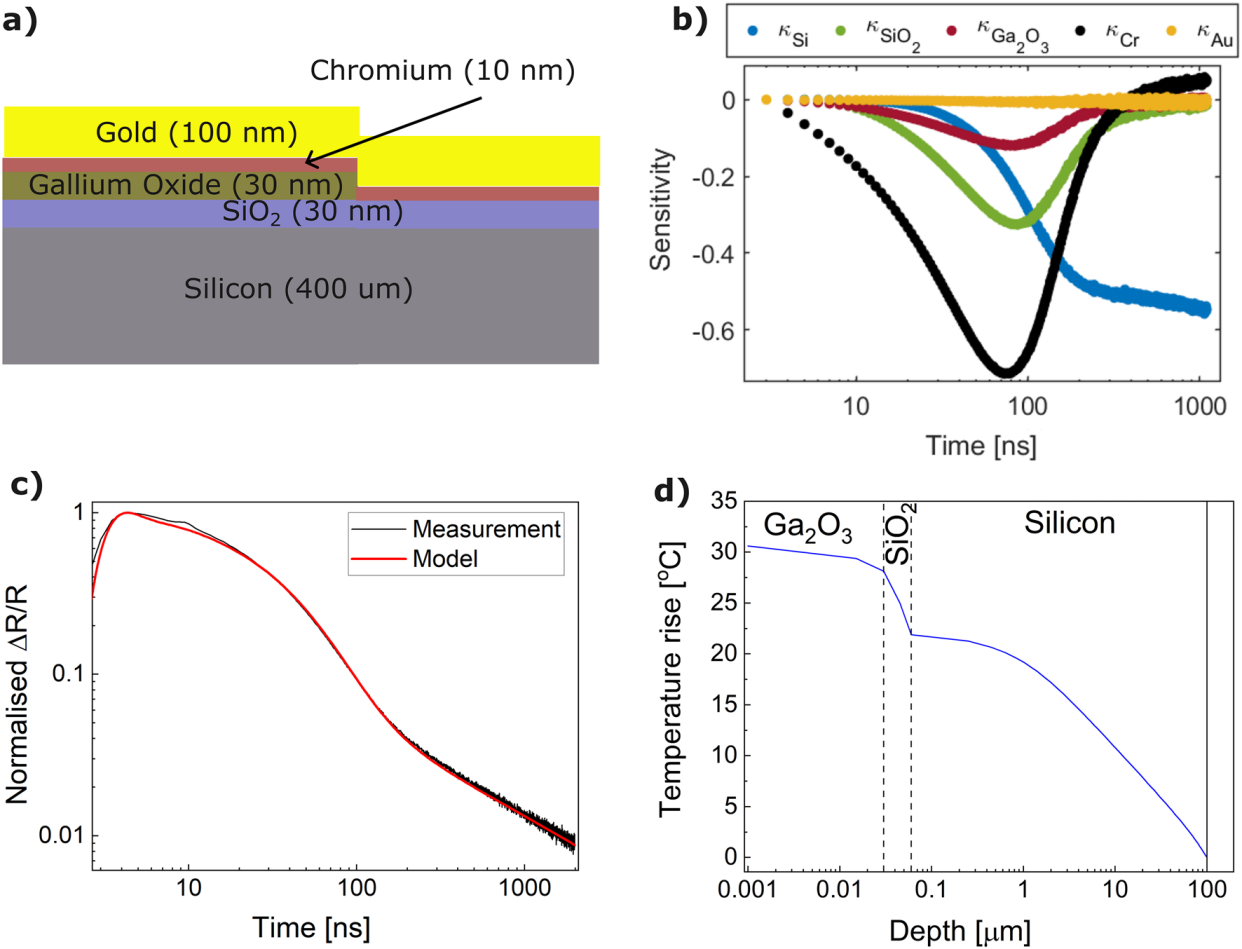
(**a**) Schematics of sample layer structure - with and without Ga$_2$O$_3$ deposition. (**b**) Plot of the fitting model’s sensitivity to the layers’ thermal conductivities as parameters. (**c**) Measured and modelled transient thermoreflectance traces for data including the Ga$_2$O$_3$ layer. (**d**) 2D FEM thermal simulation showing the $\Delta$T versus depth below a 4 $\mu$m-length, 1 Wmm$^{-1}$ heat source in the Ga$_2$O$_3$ layer.

Next, we investigate the thermal properties of the deposited thin gallium oxide film. As discussed earlier, 10 nm of Cr and 100 nm of Au were evaporated on the sample surface prior to TTR measurements. A diagram of the layers for the two areas thermoreflective transients were recorded for can be seen in Fig. 3a. Values for the out-of-plane thermal conductivity, heat capacity and density of the individual layers are presented in Table 2. The thermal conductivities used for gold and SiO$_2$ are reduced with respect to their bulk values due to their thin-film nature^38,39^. The thermal conductivity for the silicon is also reduced from its pure bulk literature value due to the effects of doping^40^. A sensitivity analysis^41^ of the thermoreflectance transient trace with respect to the thermal conductivities of the individual layers was carried out and is shown in Fig. 3b. It decouples the contributions from each layer to the overall data and indicates their relative weighting when summed up into the full transient thermal response. We note that the sensitivity to the thermal conductivity of the Ga$_2$O$_3$ is fairly low, which would imply a larger uncertainty in the fitting. On the other hand, we observe a high sensitivity to the thin Cr adhesion layer. Its thermal conductivity is first determined from fitting to the data from the bare thermal oxide on the Si substrate as $\kappa _{Cr}$=0.14±0.005 Wm$^{-1}$ K$^{-1}$, equivalent to a TBR of 7.1±0.2 m$^2$ KGW$^{-1}$. The normalised transients trace measured on the thin gallium oxide film with its fit as determined by the model is shown in Fig. 3c. With the remaining values for the layers’ thermal conductivities set (including that of the Cr layer ascertained from the dataset without any Ga$_2$O$_3$ deposition), the out-of-plane thermal conductivity of the Ga$_2$O$_3$ film is obtained as 3 ± 0.5 Wm$^{-1}$ K$^{-1}$. Taking into account the non-uniform nature of the deposition thickness, we further estimate the thermal conductivity of the film to vary between approximately 1.7 Wm$^{-1}$ K$^{-1}$ and 4.8 Wm$^{-1}$ K$^{-1}$ for thicknesses between 20 and 40 nm, respectively. This is in line with theoretical predictions for the thermal conductivity of crystalline $\beta$-Ga$_2$O$_3$ thin films (with expected values up to 4 Wm$^{-1}$ K$^{-1}$ for films of about 30 nm thickness), although lower due to its polycrystalline nature^42^.

This value, however, is twice as high as the thermal conductivity achieved from atomic layer deposition (ALD) of polycrystalline $\beta$-Ga$_2$O$_3$ film of comparable thickness onto diamond (measured as 1.5 Wm$^{-1}$ K$^{-1}$ at 30 nm thin film)^43^. Previously the thermal conductivities of polycrystalline $\beta$-Ga$_2$O$_3$ films (grown by open atmosphere annealing of GaN films) have been measured in the range between 0.34 Wm$^{-1}$ K$^{-1}$ up to 8.85 Wm$^{-1}$ K$^{-1}$ for thicknesses ranging between 12.5 nm and 895 nm respectively, which makes the result presented here on the high end of the spectrum of predicted values^44^.

**Table 2:** Parameters used for the TTR fitting.

Layer	Out-of-plane thermal conductivity [Wm$^{-1}$ K$^{-1}$]	Heat capacity [Jkg$^{-1}$ K$^{-1}]$	Density [kgm$^{-3}]$	Thickness [nm]
Au	200^38^	129^45^	19300^45^	100
Cr	0.14*	448^45^	7150^45^	10
Ga$_2$O$_3$	3*	560^45^	5880^45^	30
SiO$_2$	1.2^39^	1000^45^	2370^45^	30
Si	80^40^	700^45^	2329^45^	400,000

$^*$Obtained from fitting the experimental data.

In Fig. 3d an ANSYS 2D finite element method (FEM) simulation of the steady state temperature rise across the heterojunction is shown, using the standard and measured thermal conductivities and thicknesses given in Table 2. The simulation predicts a temperature rise of approximately 10$^{\circ }$ C across the SiO$_2$ layer from a 4 $\mu$m long 1 Wmm$^{-1}$ heat source within the Ga$_2$O$_3$ layer. By comparison, the $\Delta$T across the Ga$_2$O$_3$ layer is much smaller. This illustrates that for a typical device heat source (such as in a metal-oxide-semiconductor field-effect transistor (MOSFET)) the Ga$_2$O$_3$ layer presents a negligible thermal resistance because it is very thin. Therefore, this is a viable thermal management approach for a thin-channel transistor.

One of the aspects that contributes to the thermal resistance across an interface is the mismatch of the vibrational density of states (VDOS) between the two materials^46,47^. Among the three considered materials—Ga$_2$O$_3$, SiO$_2$ and Si, the pair with the largest VDOS overlap is Ga$_2$O$_3$/SiO$_2$, while the pair with the lowest is SiO$_2$/Si^10,48^. This suggests that a TBR between Ga$_2$O$_3$ and silicon (without SiO$_2$ as interlayer) could still be low (comparable or lower than between SiO$_2$ and Si). Of course, this should be subject to future confirmation as the phonon modes primarily responsible for interfacial thermal transport can be unique to the interface in question and are not necessarily represented in the VDOS of the individual bulk materials^49^.

In summary, electrical and thermal properties of thin-film Ga$_2$O$_3$–SiO$_2$ heterostructure were studied. We reported band offsets and out-of-plane thermal conductivity of thin-film Ga$_2$O$_3$, realized through delamination of thin passivation layers from a liquid gallium droplet onto Si with thermal oxide substrate. The estimated valence band offset of our thin film Ga$_2$O$_3$ with respect to SiO$_2$ is 0.1 eV and the predicted offset with respect to diamond is $-2.3$ eV, suggesting possibly a non-blocking interface of Ga$_2$O$_3$ with SiO$_2$ and a blocking interface with diamond. Moreover, out-of-plane thermal conductivity of thin-film Ga$_2$O$_3$ was found to be around 3 Wm$^{-1}$ K$^{-1}$, which is lower than bulk $\beta$-Ga$_2$O$_3$, although higher than what has previously been achieved for polycrystalline films of comparable thickness.

## Data Availability

The datasets generated during and/or analysed during the current study are available from the corresponding author on reasonable request.

## References

[1] Janowitz, C. *et al.* Experimental electronic structure of InO and GaO. *New J. Phys.* **13**, 085014. 10.1088/1367-2630/13/8/085014 (2011). 10.1088/1367-2630/13/8/085014

[2] Jessen, G. *et al.* Toward realization of GaO for power electronics applications. *Device Research Conference - Conference Digest* **75**, 1. 10.1109/DRC.2017.7999397 (2017). 10.1109/DRC.2017.7999397

[3] Tsao, J. Y. *et al.* Ultrawide-bandgap semiconductors: Research opportunities and challenges. *Adv. Electron. Mater.* **4**, 1600501. 10.1002/aelm.201600501 (2018). 10.1002/aelm.201600501

[4] Pearton, S. J., Ren, F., Tadjer, M. & Kim, J. Perspective: GaO for ultra-high power rectifiers and MOSFETS. *J. Appl. Phys.* **124**, 220901. 10.1063/1.5062841 (2018). 10.1063/1.5062841

[5] Reese, S. B., Remo, T., Green, J. & Zakutayev, A. How much will gallium oxide power electronics cost?. *Joule* **3**, 903–907. 10.1016/j.joule.2019.01.011 (2019). 10.1016/j.joule.2019.01.011

[6] Zhao, J. *et al.* Two-dimensional gallium oxide monolayer for gas-sensing application. *J. Phys. Chem. Lett.* **12**, 5813. 10.1021/acs.jpclett.1c01393 (2021). 34137612 10.1021/acs.jpclett.1c01393

[7] Vimalanathan, K. *et al.* High shear: In situ exfoliation of 2D gallium oxide sheets from centrifugally derived thin films of liquid gallium. *Nanoscale Adv.* **3**, 5785. 10.1039/d1na00598g (2021). 36132680 10.1039/d1na00598g PMC9419649

[8] Dejace, L., Laubeuf, N., Furfaro, I. & Lacour, S. P. Gallium-based thin films for wearable human motion sensors. *Adv. Intell. Syst.* **1**, 1900079. 10.1002/aisy.201900079 (2019). 10.1002/aisy.201900079

[9] Stepanov, S. I., Nikolaev, V. I., Bougrov, V. E. & Romanov, A. E. Gallium oxide: Properties and applications - A review. *Rev. Adv. Mater. Sci.* **44**, 63 (2016).

[10] Guo, Z. *et al.* Anisotropic thermal conductivity in single crystal -gallium oxide. *Appl. Phys. Lett.* **106**, 1. 10.1063/1.4916078 (2015). 10.1063/1.4916078

[11] Kuball, M. & Pomeroy, J. W. A review of Raman thermography for electronic and opto-electronic device measurement with submicron spatial and nanosecond temporal resolution. *IEEE Trans. Device Mater. Reliab.* **16**, 667–684. 10.1109/TDMR.2016.2617458 (2016). 10.1109/TDMR.2016.2617458

[12] Yuan, C. *et al.* Modeling and analysis for thermal management in gallium oxide field-effect transistors. *J. Appl. Phys.* **127**, 154502. 10.1063/1.5141332 (2020). 10.1063/1.5141332

[13] Ma, N. *et al.* Intrinsic electron mobility limits in -Ga2O3. *Appl. Phys. Lett.* **109**, 1. 10.1063/1.4968550 (2016). 10.1063/1.4968550

[14] Pintor-Monroy, M. I. *et al.* Tunable Electrical and Optical Properties of Nickel Oxide (NiO) Thin Films for Fully Transparent NiO-GaO p-n Junction Diodes. *ACS Appl. Mater. Interfaces.* **10**, 38159. 10.1021/acsami.8b08095 (2018). 30360100 10.1021/acsami.8b08095

[15] Li, P. *et al.* Construction of GaN/GaO p-n junction for an extremely high responsivity self-powered UV photodetector. *J. Mater. Chem. C* **5**, 10562. 10.1039/c7tc03746e (2017). 10.1039/c7tc03746e

[16] Mishra, A., Abdallah, Z., Pomeroy, J. W., Uren, M. J. & Kuball, M. Electrical and thermal performance of GaO-AlO-diamond super-junction Schottky barrier diodes. *IEEE Trans. Electron Dev.* **68**, 5055. 10.1109/TED.2021.3108120 (2021). 10.1109/TED.2021.3108120

[17] Swallow, J. E. *et al.* Transition from electron accumulation to depletion at -GaO surfaces: The role of hydrogen and the charge neutrality level. *APL Mater.* **7**, 022528. 10.1063/1.5054091 (2019). 10.1063/1.5054091

[18] Lyons, J. L. Electronic properties of GaO polymorphs. *ECS J. Solid State Sci. Technol.* **8**, Q3226. 10.1149/2.0331907jss (2019). 10.1149/2.0331907jss

[19] Lin, J. *et al.* Printing of Quasi-2D semiconducting -GaO in constructing electronic devices via room-temperature liquid metal oxide skin. *Physica Status Solidi - Rapid Res. Lett.* **13**, 1. 10.1002/pssr.201900271 (2019). 10.1002/pssr.201900271

[20] Shanks, H. R., Maycock, P. D., Sidles, P. H. & Danielson, G. C. Thermal conductivity of silicon from 300 to 1400k. *Phys. Rev.* **130**, 1743. 10.1103/PhysRev.130.1743 (1963). 10.1103/PhysRev.130.1743

[21] Zavabeti, A. *et al.* A liquid metal reaction environment for the room-temperature synthesis of atomically thin metal oxides. *Science* **358**, 332. 10.1126/science.aao4249 (2017). 29051372 10.1126/science.aao4249

[22] Martin, A., Du, C., Chang, B. & Thuo, M. Complexity and opportunities in liquid metal surface oxides. *Chem. Mater.* **32**, 9045. 10.1021/acs.chemmater.0c02047 (2020). 10.1021/acs.chemmater.0c02047

[23] Lin, R., Zheng, W., Zhang, D., Li, Y. & Huang, F. Brushed crystallized ultrathin oxides: Recrystallization and deep-ultraviolet imaging application. *ACS Appl. Electron. Mater.* **1**, 2166. 10.1021/acsaelm.9b00536 (2019). 10.1021/acsaelm.9b00536

[24] Jeong, J. *et al.* Picosecond transient thermoreflectance for thermal conductivity characterization. *Nanoscale Microscale Thermophys. Eng.* **23**, 211. 10.1080/15567265.2019.1580807 (2019). 10.1080/15567265.2019.1580807

[25] Yuan, C., Waller, W. M. & Kuball, M. Nanosecond transient thermoreflectance method for characterizing anisotropic thermal conductivity. *Rev. Sci. Instrum.* **90**, 114903. 10.1063/1.5099961 (2019). 31779394 10.1063/1.5099961

[26] Paddock, C. A. & Eesley, G. L. Transient thermoreflectance from thin metal films. *J. Appl. Phys.* **60**, 285. 10.1063/1.337642 (1986). 10.1063/1.337642 19730603

[27] Huan, Y. W. *et al.* Investigation of band alignment for hybrid 2D-MoS/3D--GaO heterojunctions with nitridation. *Nanoscale Res. Lett.* **14**, 360. 10.1186/s11671-019-3181-x (2019). 31792627 10.1186/s11671-019-3181-x PMC6889261

[28] Chen, Z. *et al.* Band alignment of GaO/Si heterojunction interface measured by X-ray photoelectron spectroscopy. *Appl. Phys. Lett.* **109**, 1. 10.1063/1.4962538 (2016). 10.1063/1.4962538

[29] Geng, S., Zhang, S. & Onishi, H. XPS applications in thin films research. *Mater. Technol.* **17**, 234. 10.1080/10667857.2002.11752992 (2002). 10.1080/10667857.2002.11752992

[30] Moulder, J. F., Stickle, W. F., Sobol, W. M. & Bomben, K. D. *Handbook of X-ray Photoelectron Spectroscopy* (Physical Electronics Division, 1992).

[31] Zatsepin, D. *et al.* Atomic structure, electronic states, and optical properties of epitaxially grown -GaO layers. *Superlattices Microstruct.* **120**, 90. 10.1016/j.spmi.2018.05.027 (2018). 10.1016/j.spmi.2018.05.027

[32] Tashmukhamedova, D. A. & Yusupjanova, M. B. Emission and optical properties of SiO/Si thin films. *Journal of Surface Investigation. X-ray, Synchrotron and Neutron Techniques* **10**, 1273, 10.1134/S1027451016050438 (2016).

[33] Keister, J. W. *et al.* Band offsets for ultrathin SiO and SiN films on Si(111) and Si(100) from photoemission spectroscopy. *J. Vacuum Sci. Technol. B: Microelectron. Nanometer Struct.* **17**, 1831. 10.1116/1.590834 (1999). 10.1116/1.590834

[34] Yadav, M. K., Mondal, A., Das, S., Sharma, S. K. & Bag, A. Impact of annealing temperature on band-alignment of PLD grown GaO/Si (100) heterointerface. *J. Alloy. Compd.* **819**, 153052. 10.1016/j.jallcom.2019.153052 (2020). 10.1016/j.jallcom.2019.153052

[35] Cook, T. E. *et al.* Measurement of the band offsets of SiO on clean n- and p-type GaN(0001). *J. Appl. Phys.* **93**, 3995. 10.1063/1.1559424 (2003). 10.1063/1.1559424

[36] Zhang, Z., Guo, Y. & Robertson, J. Atomic structure and band alignment at AlO/GaN, ScOGaN and LaO/GaN interfaces: A first-principles study. *Microelectron. Eng.* **216**, 111039. 10.1016/j.mee.2019.111039 (2019). 10.1016/j.mee.2019.111039

[37] Shi, K. *et al.* Valence band offset of GaN/diamond heterojunction measured by X-ray photoelectron spectroscopy. *Appl. Surf. Sci.* **257**, 8110. 10.1016/j.apsusc.2011.04.118 (2011). 10.1016/j.apsusc.2011.04.118

[38] Field, D. E. *et al.* Thermal characterization of direct wafer bonded Si-on-SiC. *Appl. Phys. Lett.* **120**, 113503. 10.1063/5.0080668 (2022). 10.1063/5.0080668

[39] Zhu, W., Zheng, G., Cao, S. & He, H. Thermal conductivity of amorphous SiO thin film: A molecular dynamics study. *Sci. Rep.* **8**, 1. 10.1038/s41598-018-28925-6 (2018). 30002417 10.1038/s41598-018-28925-6 PMC6043512

[40] Lee, Y. & Hwang, G. S. Mechanism of thermal conductivity suppression in doped silicon studied with nonequilibrium molecular dynamics. *Phys. Rev. B* **86**, 075202. 10.1103/PhysRevB.86.075202 (2012). 10.1103/PhysRevB.86.075202

[41] Jiang, P., Qian, X. & Yang, R. Tutorial: Time-domain thermoreflectance (TDTR) for thermal property characterization of bulk and thin film materials. *J. Appl. Phys.* **124**, 1–82. 10.1063/1.5046944 (2018). 10.1063/1.5046944

[42] Song, Y. *et al.* Thermal conductivity of -phase Ga2O3and (Al xGa1-x)2O3 heteroepitaxial thin films. *ACS Appl. Mater. Interfaces.* **13**, 38477. 10.1021/acsami.1c08506 (2021). 34370459 10.1021/acsami.1c08506

[43] Cheng, Z. *et al.* Integration of polycrystalline GaO on diamond for thermal management. *Appl. Phys. Lett.*10.1063/1.5125637 (2020). 10.1063/1.5125637

[44] Szwejkowski, C. J. *et al.* Size effects in the thermal conductivity of gallium oxide (-GaO) films grown via open-atmosphere annealing of gallium nitride. *J. Appl. Phys.*10.1063/1.4913601 (2015). 10.1063/1.4913601

[45] Haynes, W. *CRC Handbook of Chemistry and Physics, 95th Edition* (CRC Press, Taylor & Francis Group, 2015).

[46] Chen, X. K., Hu, X. Y., Jia, P., Xie, Z. X. & Liu, J. Tunable anisotropic thermal transport in porous carbon foams: The role of phonon coupling. *Int. J. Mech. Sci.* **206**, 106576. 10.1016/j.ijmecsci.2021.106576 (2021). 10.1016/j.ijmecsci.2021.106576

[47] Zhou, W. X. *et al.* Thermal conductivity of amorphous materials. *Adv. Function. Mater.*10.1002/adfm.201903829 (2020). 10.1002/adfm.201903829

[48] Deng, S. *et al.* Thermal boundary resistance measurement and analysis across SiC/SiO interface. *Appl. Phys. Lett.*10.1063/1.5111157 (2019). 10.1063/1.5111157

[49] Gordiz, K. & Henry, A. Phonon transport at interfaces: Determining the correct modes of vibration. *J. Appl. Phys.*10.1063/1.4939207 (2016). 10.1063/1.4939207

