# Author Correction: Electrical and thermal characterisation of liquid metal thin-film Ga_2_O_3_–SiO_2_ heterostructures

**DOI:** 10.1038/s41598-024-68542-0

**Published:** 2024-07-31

**Authors:** Alexander Petkov, Abhishek Mishra, Mattia Cattelan, Daniel Field, James Pomeroy, Martin Kuball

**Affiliations:** 1 HH Wills Physics Laboratory, University of Bristol, Bristol, BS8 1TL UK; 2 School of Chemistry, University of Bristol, Cantocks Close, Bristol, BS8 1TS UK; 3 Department of Chemical Sciences, University of Padova, Via Marzolo 1, 35131 Padova, Italy

Correction to: *Scientific Reports* 10.1038/s41598-023-30638-4, published online 01 March 2023

The original version of this Article contained an error in the Acknowledgements section.

“The authors acknowledge use of the University of Bristol cleanroon and NanoESCA facilities. AP acknowledges funding and support from the Engineering and Physical Sciences Research Council (EPSRC) Centre for Doctoral Training in Condensed Matter Physics (CDTCMP), Grant No. EP/L015544/1. The work of Martin Kuball was supported by the Royal Academy of Engineering through the Chair in Emerging Technologies Scheme. The authors also wish to thank Jude Lavrock for helpful discussion on XPS.”

now reads:

“The authors acknowledge use of the University of Bristol cleanroon and NanoESCA facilities. AP acknowledges funding and support from the Engineering and Physical Sciences Research Council (EPSRC) Centre for Doctoral Training in Condensed Matter Physics (CDTCMP), Grant No. EP/L015544/1, EP/X015882/1 and EP/R029393/1. The work of Martin Kuball was supported by the Royal Academy of Engineering through the Chair in Emerging Technologies Scheme. The authors also wish to thank Jude Lavrock for helpful discussion on XPS.”

The original Article has been corrected.

